# Sustainability in medical retina: the environmental impact of using aflibercept 8 mg instead of aflibercept 2 mg in treatment-naïve patients with nAMD

**DOI:** 10.1038/s41433-025-04020-9

**Published:** 2025-10-06

**Authors:** Neil Bowley, Peter Thomas, Sobha Sivaprasad, Robert M. J. Purbrick, Emily Beardmore, Rose Gilbert, Andy Clarke, Thomas J. G. Chase

**Affiliations:** 1 Royal Devon University Healthcare NHS Foundation Trust, Exeter, UK; 2 NIHR Moorfields Biomedical Research Centre, Moorfields Eye Hospital NHS Foundation Trust, London, UK; 3 UCL Institute of Ophthalmology, London, UK; 4 Sussex Eye Hospital, University Hospitals Sussex NHS Foundation Trust, Brighton, UK; 5 Bayer plc, Reading, UK

**Keywords:** Macular degeneration, Retinal diseases

## Abstract

**Background/Objectives:**

Sustainability is a major concern with the use of intravitreal therapy for neovascular age-related macular degeneration (nAMD), as case numbers rise with the ageing population. The aim of this study is to quantify the difference in the carbon emissions from factory gate to patient between aflibercept 2 mg pre-filled syringe (PFS) and aflibercept 8 mg PFS when used over the first 2 years in treatment-naïve patients with nAMD in the UK.

**Methods:**

The carbon footprint per injection was calculated by adding carbon emissions—obtained from internal corporate and published data—for packaging creation, transport, patient travel and waste disposal for each product. Results were extrapolated to a UK population using an estimate of the number of injections from real-world evidence and/or clinical trial data and the published incidence of nAMD.

**Results:**

Between factory and patient, the carbon emissions for aflibercept 2 mg PFS are approximately 2.3 kg CO_2_ per injection, compared with 2.1 kg CO_2_ for aflibercept 8 mg PFS. Using aflibercept 8 mg PFS instead of aflibercept 2 mg PFS for treatment-naïve patients with nAMD in the UK would result in ~68,000–272,000 fewer hospital visits over the first 2 years and decrease emissions by ~277,000–736,000 kg CO_2_.

**Conclusions:**

Using aflibercept 8 mg PFS instead of aflibercept 2 mg PFS in treatment-naïve patients with nAMD has benefits for the environment and National Health Service (NHS) capacity and would therefore help meet NHS sustainability goals.

## Introduction

Sustainability is a major concern within healthcare [1, 2]. Despite its importance, there has been no clear consensus on how sustainability should be defined, which has led to several different, and sometimes contradictory, recommendations for reducing the impact of healthcare practices on the environment [3]. However, carbon footprint—a measure of the greenhouse gas emissions associated with a product or process—is now widely used in commercial and industrial contexts [4] and therefore seems appropriate for evaluating sustainability within healthcare.

Healthcare emissions currently account for about 4% of global greenhouse gas emissions, and the National Health Service (NHS) has committed to net zero for all associated emissions by 2045 [1, 2]. As well as cutting down emissions controlled directly by the NHS or by commissioned pathways (e.g., buildings, water, waste and energy), carbon emissions from the supply chain must be considered. In England, the supply chain is estimated to account for 62% of the total carbon footprint of the NHS [5]; similarly, in Scotland there are approximately 8000 suppliers who provide £2.5 billion worth of goods and services to the NHS each year [6]. Personal travel (e.g., staff commutes, and patient, carer and visitor travel) is also an important contributor to NHS carbon emissions. Approximately 3.5% of all road travel in England is due to patients, visitors, and staff and suppliers to the NHS, accounting for roughly 14% of the NHS’s total emissions [1].

As ophthalmology is one of the busiest outpatient specialties in the UK [7, 8], sustainability is a particularly important consideration for healthcare professionals, integrated care boards and other relevant decision-makers working within this field. Studies have already been undertaken to identify opportunities to reduce the environmental impact of various ophthalmology practices, including cataract surgery and dry eye disease care [9, 10]. Neovascular age-related macular degeneration (nAMD) is a growing health challenge, as case numbers in the UK are expected to rise by 59% from 2015 to 2035 owing to the ageing population [11]. Demand for anti-vascular endothelial growth factor (anti-VEGF) intravitreal therapy is expected to increase accordingly, and the sustainability of different anti-VEGF agents (e.g., with regard to procurement, transport distance, packaging and injection frequency) [12–14] must be considered to reduce the environmental impact of nAMD services. Fortunately, there are many potential opportunities to decarbonise processes for procuring, reusing and disposing medicines and equipment (Table 1 [1, 14–18]).

**Table 1:** Potential opportunities for decarbonisation within the supply chain.

	Examples
Alteration of procurement processes	Use of just-in-time versus just-in-case inventory management [15]
	Introducing mandatory requirements for suppliers to decarbonise their own processes [1]
Pathway innovation to ensure efficient use of supplies	Optimising processes for decontaminating reusable equipment [16]
	Promoting circularity of plastic materials and cutting down on single-use plastic [17]
	Improving patient flow [18]
Product innovation to improve patient outcomes and reduce environmental impact	Opting for low-carbon substitutions [1]
	Using durable treatments [14]

Aflibercept (Eylea®; Bayer, Leverkusen, Germany, and Regeneron, Tarrytown, NY, USA) is indicated for the treatment of adults with nAMD in the form of a pre-filled syringe (PFS) or a vial (requiring withdrawal of solution into a sterile 1 ml syringe for delivery) for intravitreal injection [19, 20]—the former is used by the majority of healthcare providers in the UK. A high-dose (8 mg) formulation is now available [19, 20], meaning that patients with nAMD can receive less frequent injections after their initial monthly doses compared with aflibercept 2 mg while still experiencing similar clinical outcomes [21]. As aflibercept 8 mg PFS is expected to be associated with a reduced environmental impact compared with aflibercept 2 mg PFS, the aim of this study is to quantify the difference in the carbon emissions from factory gate to patient between the different formulations when used over a period of 2 years in treatment-naïve patients with nAMD in the UK.

## Materials and methods

**Fig. 1:**
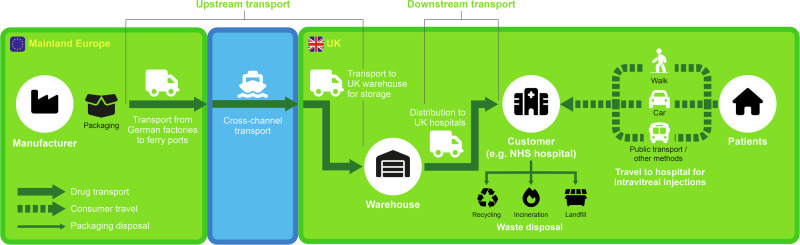
Transport of aflibercept from manufacturer to UK patients. Icons represent key stages in the delivery and disposal of aflibercept, and arrows denote different types of carbon-emitting processes.

Carbon-emitting processes required to deliver aflibercept to patients in the UK were defined (Fig. 1). It was decided that the emissions relevant to the scope of this study would start with packaging, which occurs at the factory gate.

### Carbon footprint per injection

The total carbon footprints per injection of aflibercept 8 mg PFS and aflibercept 2 mg PFS were estimated by summing the emissions for each formulation for packaging creation, upstream and downstream transport, patient travel and waste disposal.

For packaging creation, the weights of individual packaging components (provided courtesy of Bayer plc; Supplementary Table 1) were combined with published emissions for creating these materials (Supplementary Table 2) [22].

For upstream (factory to warehouse) and downstream (warehouse to customer) transport, the total annual carbon emissions were estimated using the number of shipments of aflibercept 2 mg PFS (confidential data provided courtesy of Bayer plc) and published emissions for different transport modes (Supplementary Table 2) [22]. Transport data were from the most recent 12-month period available, which was 2023/2024 (upstream) or 2022 (downstream). As downstream transport vehicles are not used exclusively for aflibercept, emissions were adjusted for the proportion of space occupied (confidential data provided courtesy of Bayer plc). To calculate carbon emissions of aflibercept 2 mg PFS per injection, the total emissions for upstream and downstream transport were divided by the total units imported and distributed during the selected time periods, respectively. As data for the transport of aflibercept 8 mg PFS were not available, emissions were estimated by adjusting the results for aflibercept 2 mg PFS according to the additional units of 8 mg PFS that fit on a single pallet. This was necessary because the aflibercept 8 mg PFS packet size is smaller than that of aflibercept 2 mg PFS (Supplementary Fig. 1).

For patient travel, carbon emissions were calculated using a published estimate of the average distance to hospital for a UK patient [23], emissions for different transport modes (Supplementary Table 2) [22] and proportional use of these transport modes in the UK. Average emissions from car travel were calculated based on the number of different vehicle types licensed in the UK at the end of 2023 [24] and their associated emissions [22] (Supplementary Table 3). Proportional use of different transport modes was estimated based on results from a 2020 online survey provided courtesy of the Macular Society [25] and UK government data [26], and emissions were adjusted to account for approximately half (50.37%) of patients being accompanied to appointments (unpublished 2024 survey data provided courtesy of the Macular Society; Supplementary Table 4).

For waste disposal, the weights of individual packaging components for each aflibercept PFS formulation (data provided courtesy of Bayer plc; Supplementary Table 1) were combined with an average of the emission factors for relevant disposal methods (closed-loop recycling, combustion and landfill) for each material type (Supplementary Table 2) [22].

### Number of injections required

For determining the number of injections of each aflibercept formulation over the first 2 years of therapy for nAMD, real-world evidence (RWE) or clinical trial data were used. RWE was identified using a literature search, which was restricted to UK studies with at least 100 patients. Only RWE studies using aflibercept 2 mg were sought, as no long-term (≥2 years) RWE was available for aflibercept 8 mg (owing to UK Medicines and Healthcare products Regulatory Agency approval in January 2024 [27]). Full details of the inclusion/exclusion criteria and search terms are provided in Supplementary Table 5. In the absence of RWE, the average number of injections for participants receiving aflibercept 8 mg was taken from the PULSAR study [21].

### Per-population modelling

The expected reduction in hospital appointments over the first 2 years in the UK was determined using the number of injections per patient over this time frame with each aflibercept formulation and an estimate of the UK incidence of nAMD. The incidence was estimated by applying the latest published incidence rate [28] to the latest (2023) UK population estimate [29]. Once the number of hospital appointments over the first 2 years in the UK with each formulation was calculated, this was combined with the estimated carbon footprint per injection to determine the projected total carbon emissions for each formulation over the first 2 years of treatment, assuming the entire eligible population was initiated on that formulation.

## Results

### Carbon footprint per injection

**Fig. 2:**
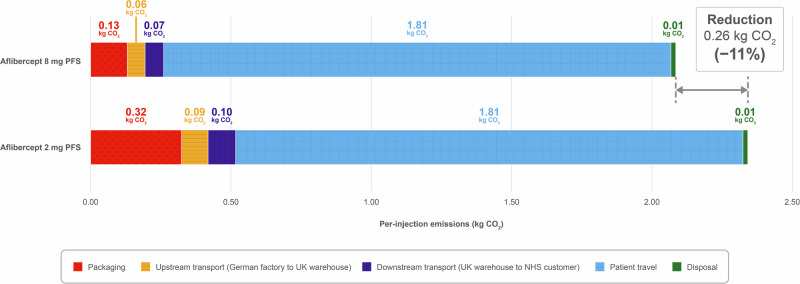
Per-injection carbon emissions of aflibercept between factory gate and UK patients. Reduction in total emissions may differ from calculations using figures shown because of rounding.

The overall carbon footprint per injection was approximately 2.3 kg CO_2_ for aflibercept 2 mg PFS and 2.1 kg CO_2_ for aflibercept 8 mg PFS (Fig. 2). The reduction in packet size of aflibercept 8 mg PFS (70 × 137 × 31 mm) relative to aflibercept 2 mg PFS (94 × 165 × 31 mm) allowed approximately 67% more units to fit on a single pallet, decreasing transport emissions by one-third. Improvements in packet composition decreased emissions for packaging creation by 60%, whereas patient travel and waste disposal emissions were unchanged or similar per injection for both formulations.

### Number of injections required

For aflibercept 2 mg PFS, the literature search for RWE identified 885 abstracts from which, following screening, 46 full publications were selected for reading. Of these, four studies were identified as providing suitable data, along with unpublished data from an investigator-initiated research project funded by Bayer plc and provided courtesy of Gruszka-Goh and Scanlon (Table 2). The main reasons for excluding other studies were that data were not specific to the UK and too few patients (<100) were included. For aflibercept 8 mg PFS, the 96-week data from the PULSAR clinical trial [21] were adjusted to 104 weeks (Supplementary Table 6) and used to provide an estimate of the number of injections over this time period. Therefore, based on the highest and lowest values from available RWE and clinical trial data, a treatment-naïve patient with nAMD was estimated to receive an average of 11.7–14.7 injections of aflibercept 2 mg and 8.7–10.2 injections of aflibercept 8 mg over the first 2 years of therapy (Table 2 [21, 30–34]).

**Table 2:** Mean number of injections of aflibercept 2 mg and aflibercept 8 mg received over the first 2 years in treatment-naïve patients with nAMD in the UK.

Real-world data^a^			
Reference	Treatment centre	*n*	Mean number of injections
Eleftheriadou et al. 2018 [30]^b^	Moorfields Eye Hospital	123	12
Lukic et al. 2021 [31]^b^	Moorfields Eye Hospital	123	12
Fu et al. 2023 [32]^c,d^	Moorfields Eye Hospital	2163	14.7
Chandra et al. 2021 [33]	Moorfields Eye Hospital	439	12.7
Gruszka-Goh and Scanlon 2024 [34]	20 UK centres	10,865	11.7
**Clinical trial data**			
**Trial name**	**Treatment arm**	***n***	**Mean number of injections**^e^
PULSAR [21]	2q8	286	13.8
	8q12	291	10.2
	8q16	292	8.7

*2q8* 2 mg every 8 weeks, *8q12* 8 mg every 12 weeks, *8q16* 8 mg every 16 weeks.

^a^Aflibercept 2 mg only.

^b^Studies provided identical data.

^c^Included only patients who had received at least seven injections in Year 1 ( ± 2 months) of treatment.

^d^Most participants were treated with fixed dosing in Year 1, followed by treat-and-extend in Year 2, but more recently treated patients were started on treat-and-extend dosing in Year 1.

^e^Adjusted to 104 weeks.

### Per-population modelling

**Fig. 3:**
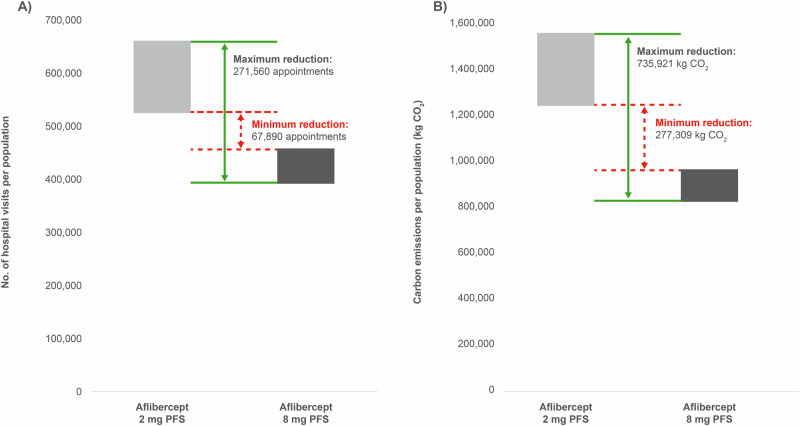
Impact of using aflibercept 8 mg PFS instead of aflibercept 2 mg PFS over the first 2 years of treatment for patients with nAMD in the UK. **A** Reduction in hospital visits and **B** Corresponding reduction in carbon emissions. Green (solid) arrows indicate the maximum expected reduction and red (dashed) arrows indicate the minimum expected reduction. Results are based on 45,260 people receiving a diagnosis of nAMD each year.

Using an incidence rate of 663 new cases of AMD per million people per year [28] and a UK population estimate of 68,265,209 [29], the annual incidence of nAMD in the UK was estimated to be 45,260. Based on this figure and assuming that one injection equated to one hospital visit, it was calculated that using aflibercept 8 mg PFS instead of aflibercept 2 mg PFS for treatment-naïve patients would result in a reduction of between approximately 68,000 and 272,000 hospital visits over the first 2 years in the UK, decreasing emissions over this time frame by approximately 277,000–736,000 kg CO_2_ (Fig. 3 and Supplementary Table 7).

## Discussion

Using aflibercept 8 mg PFS instead of aflibercept 2 mg PFS is estimated to produce between approximately 277,000 and 736,000 fewer kilograms of carbon emissions between factory gate and patient when used over the first 2 years in treatment-naïve patients with nAMD in the UK. This is equivalent to emissions saved by taking roughly 80–210 cars off the road for 2 years [22, 35], or to the carbon sequestration in a forest the size of approximately 40–110 football pitches over the same time frame [36]. Multiple factors are responsible for this reduction—for example, carbon emissions from packaging alone were reduced by about 60% because of various improvements, including a 66% reduction in the use of plastic polypropylene (PP). However, the biggest factor affecting the decrease in carbon footprint over a sustained treatment period was the reduction in patient travel, which is made possible by the improved durability associated with aflibercept 8 mg and the consequent reduction in hospital visits. Our results showed that patient travel accounted for 77%–87% of the total carbon emissions per injection; this is consistent with other similar studies carried out in France [37], New Zealand [38] and Ireland [39], which all found that patient travel accounted for a significant proportion—78%, 40% and 77%, respectively—of the total carbon footprint associated with intravitreal injections. The use of aflibercept 8 mg PFS compared with aflibercept 2 mg PFS is also likely to be associated with other environmental benefits that were beyond the scope of this study, such as decreasing the use of single-use plastics that would typically be required during each hospital visit (e.g., personal protective equipment) and savings in electricity (e.g., reduced use of fridges because of the smaller packet size of aflibercept 8 mg PFS compared with aflibercept 2 mg PFS).

The travel distance used to calculate carbon emissions for patient travel was based on the most recent available data, which estimated the average distance to a hospital to be 5.4 miles [23]. In reality, carbon emissions from patient travel will vary across the UK—for example, in rural settings, the distance travelled by patients to hospital is likely to be higher than in urban areas, with fewer public transport options. We believe that the contribution of patient travel to carbon emissions is generally likely to be greater than our results suggest, as the available data were distance as the crow flies, and land distance is generally further. The data also represent travel for emergency admissions, but patients may need to travel to a further site for specialist ophthalmology treatment. To overcome these constraints and allow readers to estimate data specific to a particular patient, a tool using individualised data is available in Supplementary Fig. 2.

We examined data from several studies to determine the number of injections of aflibercept 8 mg and aflibercept 2 mg that treatment-naïve patients with nAMD would receive over the first 2 years in the UK. The estimated number of injections for aflibercept 8 mg was available from a clinical trial setting only; however, previous evidence indicates that aflibercept data from RWE is comparable to results seen in pivotal clinical trials [40–42]. There was no clear rationale for selecting any one study as a data source, so the highest and lowest values across all suitable literature were used to account for heterogeneity. As clinical practice varies between treatment centres, we thought it would be most realistic to provide our results as a range rather than a single value. Similarly, we used a UK average incidence rate of nAMD, but in reality the rate will vary between regions (e.g., because of regional demographic variation).

Sustainability benefits associated with using aflibercept 8 mg instead of aflibercept 2 mg may be even greater over a longer period (e.g., 4 years) than captured in this study. Aflibercept 8 mg has a UK licence for treatment intervals of up to 5 months, but the injection numbers for aflibercept 8 mg in this study (adjusted average: 8.7–10.2 injections over the first 2 years) are based on data from the PULSAR trial, in which treatment intervals were limited to every 8, 12 or 16 weeks in Year 1 [19, 43].

As well as reducing injection frequency, there are other strategies that could potentially help improve the sustainability of ophthalmology practices. Travel could be minimised by moving delivery of care to small, local treatment centres and by using digital technology for remote monitoring and screening of nAMD and virtual consultations [13, 44, 45]. The evidence base for normal infection control procedures—for example, use of sterile intravitreal packs, sterile gloves and prophylactic topical antibiotics—could be evaluated to identify opportunities for waste reduction [13]. It is also important to underpin these changes with training programmes for healthcare professionals that promote sustainable healthcare as a common approach [45].

In addition to environmental benefits, use of a durable anti-VEGF treatment also has other advantages. The NHS continues to operate under considerable strain and reducing this pressure is an important consideration [46, 47]. Examining the impact on staffing is beyond the scope of this study, but it is possible that a reduction in hospital visits for patients could also have benefits for staff capacity. Moreover, durability has an important impact on cost-effectiveness as well as sustainability, and this should be considered when using anti-VEGF treatment [48]. With ophthalmology being one of the busiest outpatient specialties in the UK [7, 8], ophthalmology clinics are already under considerable pressure from the burden of patients requiring hospital visits for intravitreal injections. With an ageing population [11], costs could rise owing to services being over capacity and consequently reliant on extra weekend and evening clinics. A drug that requires less frequent injections may be more cost-effective overall than one that requires more frequent injections when the wider costs of running an nAMD service clinic are considered, particularly when services are under strain [48]. Finally, as well as benefits for NHS capacity, a reduction in hospital visits would decrease the burden of treatment for patients and caregivers.

Our study has some limitations. As the data underpinning the calculations pertain specifically to aflibercept 8 mg PFS compared with aflibercept 2 mg PFS, the considerations and results may not be applicable to other longer-acting anti-VEGF agents. The details of the manufacturing process were not available to the authors; hence, we focused on carbon emissions between factory and patient only. This design was consistent with a similar study in Ireland [39]. Additional hospital visits exclusively for monitoring were not captured, as accurate information about the proportion of NHS Trusts that run decoupled services could not be found; this practice was also not considered common enough to warrant making assumptions that could compromise the validity of the calculations. Similarly, staff travel was not included in our calculations because staff commute to a hospital would not necessarily be changed by the injection frequency of aflibercept and this would have added ambiguity to the results. Our calculations also omitted building usage because this was found to be negligible, accounting for only 5% of carbon emissions in a similar study [39]. We did not have accurate data about waste disposal methods, so we used an average of the emission factors for incineration, landfill and recycling. We acknowledge that the proportional use of different methods is likely to vary by NHS Trust, but as incineration and recycling are associated with the same emissions, and waste disposal accounted for less than 0.5% of total emissions in any case, any change to this figure would have a negligible impact on the final result and would not change the difference in emissions between the two formulations. Finally, the data in our study are limited to the first 2 years of treatment, as predictions beyond this time period are difficult to quantify because of the lack of clinical trials and real-world studies with data from a longer time period.

In conclusion, our study indicates that using aflibercept 8 mg PFS instead of aflibercept 2 mg PFS in treatment-naïve patients with nAMD has benefits for the environment and NHS capacity over a period of 2 years and would therefore help improve sustainability within healthcare. Although previous studies quantified the carbon footprint of specific aspects of an nAMD service in different ways, our study takes a solution-focused approach by assessing the reduction in carbon footprint of using a more durable anti-VEGF treatment. The results also indicate that using durable agents in other retinal diseases (e.g., diabetic macular oedema) would also have a positive impact on the environment and NHS capacity. It is important to emphasise that these results should be interpreted with caution, owing to the many assumptions and estimations that were necessary, and a long-term prospective study is needed to reinforce these findings. Nonetheless, it is clear that balancing efficacy, accessibility and sustainability across healthcare will be key to meeting the healthcare needs of the UK population in the future.

Supplementary information is available on *Eye*’s website.

## Summary

### What was known before

- The treatment course for neovascular age-related macular degeneration (nAMD) by intravitreal injection requires frequent patient appointments, with a considerable burden in terms of patient travel and National Health Service (NHS) services, resulting in a significant environmental impact.
- Previous studies have attempted to quantify the carbon footprint associated with a nAMD service in different ways—for example, emissions from intravitreal injections in Ireland or France or from a day at an ophthalmology department in New Zealand.
- Using durable treatments offers promise for alleviating the burden related to frequent patient visits and, consequently, the environmental impact.

### What this study adds

- This study evaluates the total environmental impact of the first 2 years of anti-vascular endothelial growth factor injections for a patient with nAMD while also providing a comparison with a more durable treatment.
- Evaluating the carbon emissions, access to internal corporate data about the transport logistics of aflibercept, the units imported and distributed annually, and the packaging components allowed an in-depth and accurate calculation of emissions between factory gate and patient.
- Our study provides data specific to the UK population and is relevant to NHS sustainability goals.

## Supplementary information

Supplementary Table 1. Weight of packaging components of aflibercept 2 mg and aflibercept 8 mg PFS.

Supplementary Table 2. Sources of emission factors.

Supplementary Table 3. Calculation of average emission factors for car transport.

Supplementary Table 4. Proportional use of different transport modes for travel to hospital and associated emissions.

Supplementary Table 5. Literature search criteria.

Supplementary Table 6. Average number of injections for participants receiving aflibercept 2 mg and 8 mg in the PULSAR study.

Supplementary Table 7. Calculations for the reduction in hospital visits and corresponding carbon emissions over the first 2 years in the UK from using aflibercept 8 mg PFS instead of aflibercept 2 mg PFS in treatment-naïve patients with nAMD.

Supplementary Fig. 1 Units of aflibercept 2 mg and 8 mg PFS per pallet.

Supplementary Fig. 2 Individualised carbon emissions between factory gate and consumer for A) aflibercept 2 mg PFS and B) aflibercept 8 mg PFS.

## Data Availability

The datasets with the number of shipments of aflibercept 2 mg in upstream and downstream transport analysed during the current study are not publicly available owing to being commercially sensitive data (Bayer plc), but they are available from the corresponding author on reasonable request. All other data generated or analysed during this study are included or referenced in this published article and its Supplementary Information files.
