# Author Correction: Sustainability in medical retina: the environmental impact of using aflibercept 8 mg instead of aflibercept 2 mg in treatment-naïve patients with nAMD

**DOI:** 10.1038/s41433-026-04663-2

**Published:** 2026-07-10

**Authors:** Neil Bowley, Peter Thomas, Sobha Sivaprasad, Robert M. J. Purbrick, Emily Beardmore, Rose Gilbert, Andy Clarke, Thomas J. G. Chase

**Affiliations:** 1 Royal Devon University Healthcare NHS Foundation Trust, Exeter, UK; 2 NIHR Moorfields Biomedical Research Centre, Moorfields Eye Hospital NHS Foundation Trust, London, UK; 3 UCL Institute of Ophthalmology, London, UK; 4 Sussex Eye Hospital, University Hospitals Sussex NHS Foundation Trust, Brighton, UK; 5 Bayer plc, Reading, UK

**Keywords:** Macular degeneration, Retinal diseases

Correction to: *Eye*

10.1038/s41433-025-04020-9, published 06 October 2025

In this article, the current text has been published erroneously: ‘The reduction in packet size of aflibercept 8 mg PFS (70 × 137 × 31 mm) relative to aflibercept 2 mg PFS (94 × 135 × 31 mm) allowed approximately 67% more units to fit on a single pallet, decreasing transport emissions by one-third.’

It should read:

'The reduction in packet size of aflibercept 8 mg PFS (70 × 137 × 31 mm) relative to aflibercept 2 mg PFS (94 × 165 × 31 mm) allowed approximately 67% more units to fit on a single pallet, decreasing transport emissions by one-third.’

The original article has been corrected.

