# Supplementary material for: Sustainability in medical retina: the environmental impact of using aflibercept 8 mg instead of aflibercept 2 mg in treatment-naïve patients with nAMD
Source: Eye (Lond). 2025 Oct 6;39(17):3160–6. doi: 10.1038/s41433-025-04020-9 (PMC12624108; doi:10.1038/s41433-025-04020-9)
Supplement: Supplementary file 1 — Supplementary Table 1. Weight of packaging components of aflibercept 2 mg and aflibercept 8 mg PFS. [file 41433_2025_4020_MOESM1_ESM.docx]

**Supplementary Table 1.** Weight of packaging components of aflibercept 2 mg and aflibercept 8 mg PFS.

|  | **Aflibercept PFS packet** | | | | | |
| --- | --- | --- | --- | --- | --- | --- |
|  | **2 mg** | | | **8 mg** | | |
|  | **Primary** | **Secondary** | **Total** | **Primary** | **Secondary** | **Total** |
| Paper/Card | 0.0 g | 24.3 g | 24.3 g | 8.6 g | 18.0 g | 26.6 g |
| Glass | 3.3 g | 0.0 g | 3.3 g | 0.0 g | 0.0 g | 0.0 g |
| Plastic PP | 3.7 g | 107.0 g | 110.7 g | 35.1 g | 2.0 g | 37.1 g |
| Rubber | 0.6 g | 0.0 g | 0.6 g | 0.0 g | 0.0 g | 0.0 g |
| **TOTAL** | **7.6 g** | **131.3 g** | **138.9 g** | **43.7 g** | **20.0 g** | **63.7 g** |

*PFS* pre-filled syringe, *PP* polypropylene.
