# Supplementary material for: Sustainability in medical retina: the environmental impact of using aflibercept 8 mg instead of aflibercept 2 mg in treatment-naïve patients with nAMD
Source: Eye (Lond). 2025 Oct 6;39(17):3160–6. doi: 10.1038/s41433-025-04020-9 (PMC12624108; doi:10.1038/s41433-025-04020-9)
Supplement: Supplementary file 2 — Supplementary Table 2. Sources of emission factors. [file 41433_2025_4020_MOESM2_ESM.docx]

**Supplementary Table 2.** Sources of emission factors [22].

| **Emission factor** | **Source description** | **Application** |
| --- | --- | --- |
| *Transport* | | |
| 1.86 | 100% laden refrigerated HGV (all diesel), kg CO_2_e per mile | Return journeys of vehicles transporting aflibercept from German factory to UK warehouse (excluding channel crossing) and from UK warehouse to NHS customers |
| 0.03 | 4000+ CEU vehicle transport cargo ship, kg CO_2_e per tonne.km* | - Return journey for cross-channel ferry transport of aflibercept - Emissions per shipping container, not per ferry |
| 0.21 | Regular taxi, kg CO_2_e per km | - Return journey for patient transport to hospital by taxi (converted to miles) - Emissions for entire vehicle, not per passenger |
| 0.11 | Average local bus, kg CO_2_e per passenger.km^†^ | - Return journey for one patient travelling to hospital by bus (converted to miles) - Emissions per passenger, not for entire bus |
| 0.02 | Average based on kg CO_2_e per passenger.km^†^ for National Rail (0.04) and London Underground (0.03) converted to miles | - Return journey for one patient travelling to hospital by rail (converted to miles) - Emissions per passenger, not for entire train |
| 0.03 | Light rail and tram, kg CO_2_e per passenger.km^†^ | - Return journey for one patient travelling to hospital by light rail or tram (converted to miles) - Emissions per passenger, not for entire train/tram |
| 0.18 | Average motorbike, kg CO_2_e per mile | - Return journey for one motorbike used to transport a patient to hospital - Emissions for entire motorbike, not per passenger |
| 0.26 | Average for cars weighted by 2023 proportional registration of different car types in the UK (see **Supplementary Table 3**), kg CO_2_e per mile | - Return journey for one family or hospital car used to transport a patient to hospital - Emissions for entire vehicle, not per passenger |
| 0.28 | Average for MPVs weighted by 2023 proportional registration of different car types in the UK (see **Supplementary Table 3**), kg CO_2_e per mile | - Return journey for one minibus (e.g. community/patient transport) used to transport a patient to hospital - Emissions for entire vehicle, not per passenger |
| 0.00 | N/A (assumed to be battery powered) | - Return journey for one mobility scooter to transport a patient to hospital |
| 0.00 | N/A (assumed to be battery powered) | - Return journey for one motorised wheelchair to transport a patient to hospital |
| *Packaging* | | |
| 1339.32 | Primary material use; paper and board: paper, kg CO_2_e per tonne | Creation of paper/card component of product packaging |
| 1402.77 | Primary material use; glass, kg CO_2_e per tonne | Creation of glass component of product packaging |

**Supplementary Table 2.** Sources of emission factors [22] (cont.).

| **Emission factor** | **Source description** | **Application** |
| --- | --- | --- |
| *Packaging (cont.)* | | |
| 2568.59 | Primary material use; plastics: PP (incl. forming), kg CO_2_e per tonne | Creation of plastic PP component of product packaging |
| 3335.57 | Primary material use; tyres^‡^, kg CO_2_e per tonne | Creation of rubber component of product packaging |
| *Waste disposal by recycling* | | |
| 6.41 | Closed-loop recycling; paper and board: paper, kg CO_2_e per tonne | Recycling of paper/card component of product packaging |
| 6.41 | Closed-loop recycling; glass, kg CO_2_e per tonne | Recycling of glass component of product packaging |
| 6.41 | Closed-loop recycling; plastics: PP (incl. forming), kg CO_2_e per tonne | Recycling of plastic PP component of product packaging |
| 6.41 | Closed-loop recycling; tyres^‡^, kg CO_2_e per tonne | Recycling of rubber component of product packaging |
| *Waste disposal by incineration* | | |
| 6.41 | Combustion; paper and board: paper, kg CO_2_e per tonne | Incineration of paper/card component of product packaging |
| 6.41 | Combustion; glass, kg CO_2_e per tonne | Incineration of glass component of product packaging |
| 6.41 | Combustion; plastics: PP (incl. forming), kg CO_2_e per tonne | Incineration of plastic PP component of product packaging |
| 6.41 | Combustion; household residual waste^§^, kg CO_2_e per tonne | Incineration of rubber component of product packaging |
| *Waste disposal by landfill* | | |
| 1,164.39 | Landfill; paper and board: paper, kg CO_2_e per tonne | Landfill disposal of paper/card component of product packaging |
| 8.88 | Landfill; glass, kg CO_2_e per tonne | Landfill disposal of glass component of product packaging |
| 8.88 | Landfill; plastics: PP (incl. forming), kg CO_2_e per tonne | Landfill disposal of plastic PP component of product packaging |
| 497.04 | Landfill; household residual waste^§^, kg CO_2_e per tonne | Landfill disposal of rubber component of product packaging |

*CEU* car equivalent units, *CO_2_e* carbon dioxide emissions, *HGV* heavy goods vehicle, *MPV* multiperson vehicle, *N/A* not applicable, *NHS* National Health Service, *PP* polypropylene.
*An equivalent measure of one tonne of transported goods over one km.
^†^The distance travelled by individual passengers in a transport mode.
^‡^Used as a value was not provided for rubber.
^§^Used as values were not provided for rubber or tyres.
