# Supplementary material for: Sustainability in medical retina: the environmental impact of using aflibercept 8 mg instead of aflibercept 2 mg in treatment-naïve patients with nAMD
Source: Eye (Lond). 2025 Oct 6;39(17):3160–6. doi: 10.1038/s41433-025-04020-9 (PMC12624108; doi:10.1038/s41433-025-04020-9)
Supplement: Supplementary file 3 — Supplementary Table 3. Calculation of average emission factors for car transport. [file 41433_2025_4020_MOESM3_ESM.docx]

**Supplementary Table 3.** Calculation of average emission factors for car transport.

|  | **No. of cars in the UK in 2023, millions [24]** | | **Emission factor [22]** | |
| --- | --- | --- | --- | --- |
|  | **Subtypes** | **Total** | **Average car** | **MPV** |
| **Petrol** | N/A | 18.74 | 0.26 | 0.29 |
| **Diesel** | N/A | 10.67 | 0.27 | 0.29 |
| **EV** | N/A | 0.92 | 0.00 | 0.00 |
| **Hybrid – Electric** | | | | |
| Petrol | 1.57 | 1.66 | 0.20 | N/A |
| Diesel | 0.10 |  |  |  |
| **Hybrid – Plug-in electric** | | | | |
| Petrol | 0.51 | 0.52 | 0.15 | 0.13 |
| Diesel | 0.01 |  |  |  |
| **Weighted average** | | | **0.26** | **0.28** |

*EV* electric vehicle, *MPV* multiperson vehicle, *N/A* not available.
