# Supplementary material for: Sustainability in medical retina: the environmental impact of using aflibercept 8 mg instead of aflibercept 2 mg in treatment-naïve patients with nAMD
Source: Eye (Lond). 2025 Oct 6;39(17):3160–6. doi: 10.1038/s41433-025-04020-9 (PMC12624108; doi:10.1038/s41433-025-04020-9)
Supplement: Supplementary file 4 — Supplementary Table 4. Proportional use of different transport modes for travel to hospital and associated emissions. [file 41433_2025_4020_MOESM4_ESM.docx]

**Supplementary Table 4.** Proportional use of different transport modes for travel to hospital and associated emissions.

|  | **Proportion of trips** | **Emission factor**^‡^ | **kg CO_2_e per trip^∥^** | | | **Proportion of emissions** |
| --- | --- | --- | --- | --- | --- | --- |
|  |  |  | **Patient** | **Carer^#^** | **Total** |  |
| **Walk** | 5%* | 0.00 | 0 | 0 | 0 | 0 |
| **Car** | 55%* | 0.26 | 1.52 | Included | 1.52 | 84% |
| **Public transport** |  |  |  |  |  |  |
| - Taxi | 9%***** | 0.13 | 0.13 | Included | 0.13 | 7% |
| - Bus | 12%**^†^** | 0.07 | 0.09 | 0.04 | 0.13 | 7% |
| - Rail | 9%**^†^** | 0.02 | 0.02 | 0.01 | 0.03 | 2% |
| - Tram and light rail | 3%**^†^** | 0.02 | 0.01 | <0.01 | 0.01 | 0 |
| **Other** | 7%* | 0.14^§^ | 0.11 | 0.05 | 0.16 | 9% |
|  |  |  |  | **TOTAL** | **1.81** | **100%** |

*CO_2_e* carbon dioxide emissions, *MPV* multiperson vehicle.

*Figures from a 2020 UK online survey [25] provided courtesy of the Macular Society.

^†^The Macular Society 2020 UK online survey [25] reported that 24% of journeys were by public transport. No further breakdown was available, so it was assumed the ratio of different public transport modes was consistent with the latest (2023) UK government data [26], in which public transport use accounted for 8% of journeys: 4% bus, 3% rail (surface rail and London Underground) and 1% other (taxi, minicab, air, ferry, light rail and tram). As taxi use was accounted for separately and air/ferries would not be used for hospital travel by the majority of the population, light rail and trams were assumed to account for all other public transport use in this case.

^‡^See **Supplementary Table 2** for source descriptions.

^§^The Macular Society 2020 UK online survey [25] reported that 7% of all journeys were not by car, taxi or public transport. No further breakdown was available, so it was assumed that these journeys were by other private transport methods. Of the methods listed under this category in UK government transport data, those applicable for an elderly population attending a routine hospital appointment were motorcycles, mobility scooters, motorised wheelchairs, hospital cars and private hire buses [26]. An average of the emission factors for these transport modes was used (0.18, 0.00, 0.00, 0.26 and 0.28, respectively; see **Supplementary Table 2**) [22]. Note that a private hire bus was assumed to be a minibus (e.g. for community/patient transport), but an emission factor for this specific vehicle type was not available in UK government data. Therefore, an average emission factor for an MPV based on the 2023 proportional registration of different car types in the UK [24] and associated emission factors [22] was used (see **Supplementary Table 3**).

^∥^Based on a return journey to a hospital at an average distance of 5.4 miles [23].

^#^Results from a 2024 survey, provided courtesy of the Macular Society, showed that 50.37% of patients were always accompanied for each visit, 42% were unaccompanied and 7.62% were sometimes accompanied. The lowest value of 50.37% was used as a conservative estimate of the proportion of patients that would be accompanied by one carer. Emissions for car or taxi journeys are per vehicle [22] and therefore did not require adjustment.
