# Supplementary material for: Sustainability in medical retina: the environmental impact of using aflibercept 8 mg instead of aflibercept 2 mg in treatment-naïve patients with nAMD
Source: Eye (Lond). 2025 Oct 6;39(17):3160–6. doi: 10.1038/s41433-025-04020-9 (PMC12624108; doi:10.1038/s41433-025-04020-9)
Supplement: Supplementary file 5 — Supplementary Table 5. Literature search criteria. [file 41433_2025_4020_MOESM5_ESM.docx]

**Supplementary Table 5.** Literature search criteria.

| **Inclusion criteria** | - RWE in the UK - Aflibercept 2 mg use - Treatment-naïve patients - nAMD patients |
| --- | --- |
| **Exclusion criteria** | - Non-English language - Not a UK-based study - Clinical trial not RWE - Animal trial - <100 patients - Switch patients - <96-week outcome - Review article |
| **Search terms (on PubMed)** | ((Aflibercept[Title/Abstract] OR aflibercept[MeSH Terms]) AND (Age-related Macular Degeneration[Title/Abstract] OR AMD[Title/Abstract]) AND (outcomes[Title/Abstract] OR efficacy[Title/Abstract] OR safety[Title/Abstract] OR injection number)) |

*AMD* age-related macular degeneration, *nAMD* neovascular age-related macular degeneration, *RWE* real‑world evidence.
