# Supplementary material for: Sustainability in medical retina: the environmental impact of using aflibercept 8 mg instead of aflibercept 2 mg in treatment-naïve patients with nAMD
Source: Eye (Lond). 2025 Oct 6;39(17):3160–6. doi: 10.1038/s41433-025-04020-9 (PMC12624108; doi:10.1038/s41433-025-04020-9)
Supplement: Supplementary file 6 — Supplementary Table 6. Average number of injections for participants receiving aflibercept 2 mg and 8 mg in the PULSAR study. [file 41433_2025_4020_MOESM6_ESM.docx]

**Supplementary Table 6.** Average number of injections for participants receiving aflibercept 2 mg and 8 mg in the PULSAR study.

|  | **Treatment group** | | |
| --- | --- | --- | --- |
| **No. of injections** | **2q8** | **8q12** | **8q16** |
| *a*) Week 96 data [21] | 12.8 | 9.6 | 8.2 |
| *b*) Week 48 data [43] | 6.9 | 6.1 | 5.2 |
| *c*) Between Week 48 and Week 96  (= *a* − *b*) | 5.9 | 3.5 | 3.0 |
| *d*) Between Week 48 and Week 104  (= *c* ÷ 48 × 56) | 6.9 | 4.1 | 3.5 |
| Between Week 0 and Week 104  (= *b* + *d*) | 13.8 | 10.2 | 8.7 |

*2q8* 2 mg every 8 weeks, *8q12* 8 mg every 12 weeks, *8q16* 8 mg every 16 weeks.
