# Supplementary material for: Sustainability in medical retina: the environmental impact of using aflibercept 8 mg instead of aflibercept 2 mg in treatment-naïve patients with nAMD
Source: Eye (Lond). 2025 Oct 6;39(17):3160–6. doi: 10.1038/s41433-025-04020-9 (PMC12624108; doi:10.1038/s41433-025-04020-9)
Supplement: Supplementary file 7 — Supplementary Table 7. Calculations for the reduction in hospital visits and corresponding carbon emissions over the first 2 years in the UK from using aflibercept 8 mg PFS instead of aflibercept 2 mg PFS in treatment-naïve patients with nAMD. [file 41433_2025_4020_MOESM7_ESM.docx]

**Supplementary Table 7.** Calculations for the reduction in hospital visits and corresponding carbon emissions over the first 2 years in the UK from using aflibercept 8 mg PFS instead of aflibercept 2 mg PFS in treatment-naïve patients with nAMD.*

|  | | **Aflibercept 2 mg PFS** | **Aflibercept 8 mg PFS** |
| --- | --- | --- | --- |
| **No. of hospital visits** | | | |
| Per patient | Maximum | 14.7 | 10.2 |
|  | Minimum | 11.7 | 8.7 |
| Per population^†^ | Maximum | 665,322 | 461,652 |
|  | Minimum | 529,542 | 393,762 |
| Overall reduction | Maximum^‡^ | 271,560 | |
|  | Minimum^§^ | 67,890 | |
| **kg CO_2_e** | | | |
| Per injection | | 2.98 | 2.72 |
| Per population^∥^ | Maximum | 1,555,117 | 960,438 |
|  | Minimum | 1,237,746 | 819,197 |
| Overall reduction | Maximum^‡^ | 735,921 | |
|  | Minimum^§^ | 277,309 | |

*CO_2_e* carbon dioxide emissions, *nAMD* neovascular age-related macular degeneration, *PFS* pre-filled syringe.

*Results are based on 45,260 people receiving a diagnosis of nAMD each year.

^†^Number of visits per patient multiplied by the population diagnosed with nAMD in the UK each year.

^‡^Minimum per‑population estimate for aflibercept 8 mg PFS subtracted from the maximum per‑population estimate for aflibercept 2 mg PFS.

^§^Maximum per‑population estimate for aflibercept 8 mg PFS subtracted from the minimum per‑population estimate for aflibercept 2 mg PFS.

^∥^Emissions per injection multiplied by the number of hospital visits per population.
