# Supplementary material for: Sustainability in medical retina: the environmental impact of using aflibercept 8 mg instead of aflibercept 2 mg in treatment-naïve patients with nAMD
Source: Eye (Lond). 2025 Oct 6;39(17):3160–6. doi: 10.1038/s41433-025-04020-9 (PMC12624108; doi:10.1038/s41433-025-04020-9)
Supplement: Supplementary file 8 — Supplementary Fig. 1 Units of aflibercept 2 mg and 8 mg PFS per pallet. [file 41433_2025_4020_MOESM8_ESM.docx]

**Supplementary Fig. 1** Units of aflibercept 2 mg and 8 mg PFS per pallet.


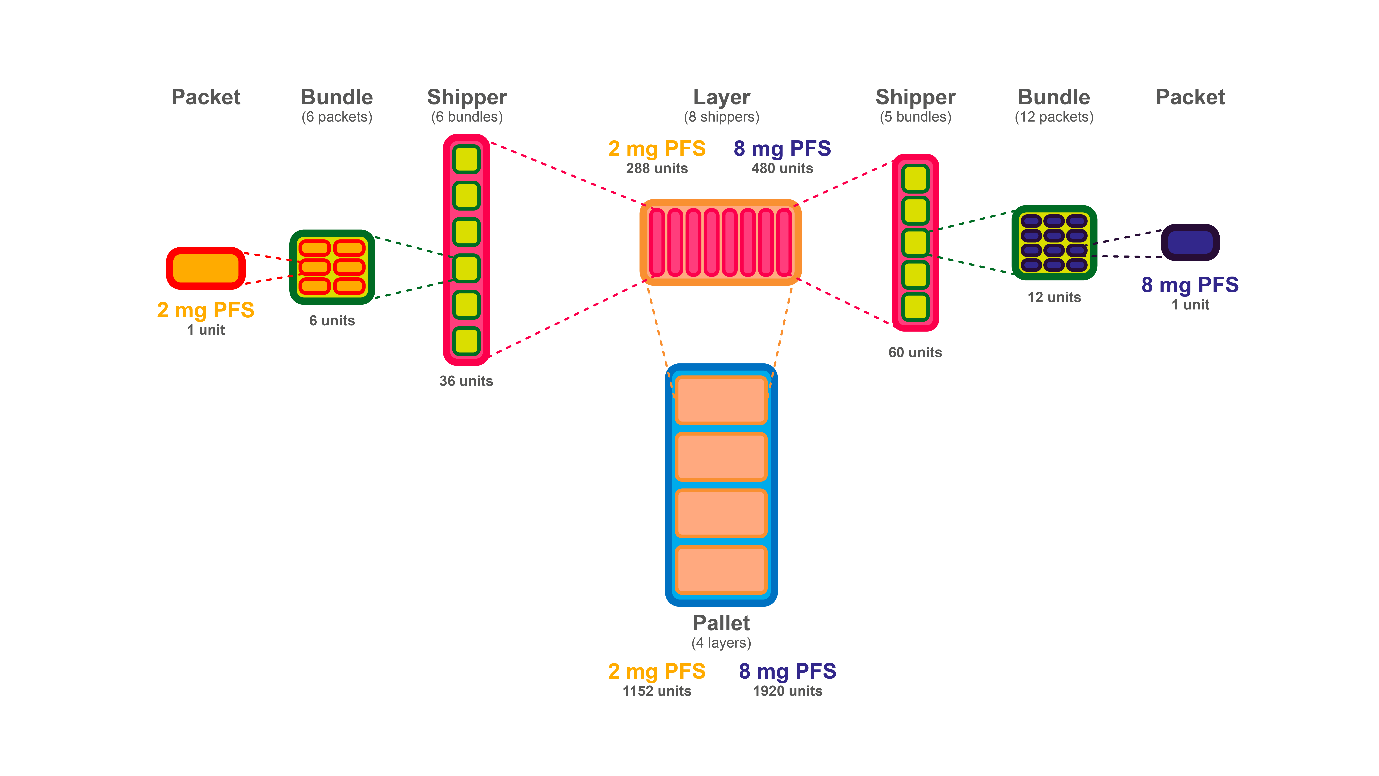


*PFS* pre-filled syringe.

Note that this depiction has been created for the purposes of illustrating the difference in the number of units per pallet between aflibercept 2 mg and 8 mg PFS and may not accurately reflect the way in which the packets are stacked.
