# Supplementary material for: Sustainability in medical retina: the environmental impact of using aflibercept 8 mg instead of aflibercept 2 mg in treatment-naïve patients with nAMD
Source: Eye (Lond). 2025 Oct 6;39(17):3160–6. doi: 10.1038/s41433-025-04020-9 (PMC12624108; doi:10.1038/s41433-025-04020-9)
Supplement: Supplementary file 9 — Supplementary Fig. 2 Individualised carbon emissions between factory gate and consumer for A) aflibercept 2 mg PFS and B) aflibercept 8 mg PFS. [file 41433_2025_4020_MOESM9_ESM.docx]

**Supplementary Fig. 2** Individualised carbon emissions between factory gate and consumer for A) aflibercept 2 mg PFS and B) aflibercept 8 mg PFS.


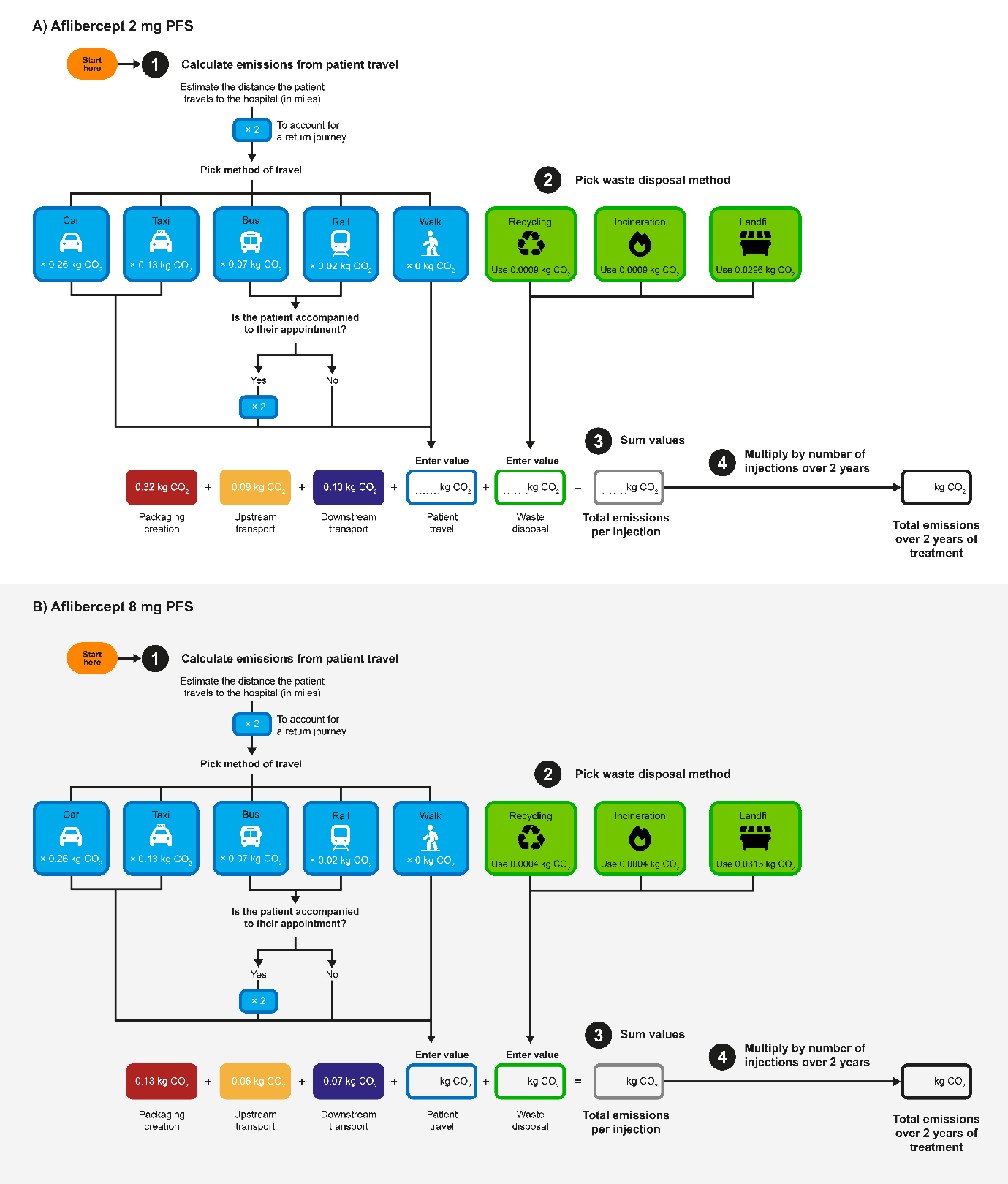


*PFS* pre-filled syringe.

The values shown in the figure are specific to the product formulations shown, and the total emissions are for the stages between factory gate and patient only. Development of this tool was funded by Bayer plc, UK.
